# Unraveling the molecular mechanisms of Fufangduzhong formula in alleviating high-fat diet-induced non-alcoholic fatty liver disease in mice

**DOI:** 10.3389/fphar.2025.1542143

**Published:** 2025-03-12

**Authors:** Yu Mou, Yao Tang, Xiuyan Zheng, Xiang Liu, Xuemei Wu, Hongji Wang, Jie Zeng, Qing Rao, Yaacov Ben-David, Yanmei Li, Lei Huang

**Affiliations:** ^1^ State Key Laboratory of Discovery and Utilization of Functional Components in Traditional Chinese Medicine, Guizhou Medical University, Guiyang, China; ^2^ Natural Products Research Center of Guizhou Province, Guiyang, China; ^3^ School of Pharmaceutical Sciences, Guizhou Medical University, Guiyang, China; ^4^ School of Basic Medical, Guizhou Medical University, Guiyang, China; ^5^ Guizhou Institute of Integrated Agriculture Development, Guiyang, China

**Keywords:** non-alcoholic fatty liver disease, Fufangduzhong formula, transcriptomics, serum metabolomics, lipid metabolism

## Abstract

**Background:**

Non-alcoholic fatty liver disease (NAFLD) is a common chronic liver disease, characterized by hepatic lipid accumulation. The Fufangduzhong formula (FFDZ) is a traditional Chinese medicine (TCM) formulation composed of *Eucommia ulmoides* Oliv.*, Leonurus artemisia* (Lour.) S. Y. Hu, *Prunella vulgaris* Linn*, Uncariarhynchophylla* (Miq.) Miq. ex Havil., *and Scutellaria baicalensis* Georgi. It has demonstrated hepatoprotective effects and the ability to reduce lipid accumulation. However, its mechanisms against NAFLD remain unclear.

**Methods:**

UPLC-MS/MS was used to identify FFDZ metabolites. C57BL/6J mice were fed a high-fat diet (HFD) supplemented with or without FFDZ (HFD+L, 0.45 g/kg/d; HFD+H, 0.9 g/kg/d) for 12 weeks. Biochemical indicators and histopathological observations were utilized to assess the extent of metabolic homeostasis disorder and hepatic steatosis. An analysis of differentially expressed genes and regulated signaling pathways was conducted using hepatic transcriptomics. Metabolomics analysis was performed to investigate the significantly changed endogenous metabolites associated with NAFLD in mice serum using UPLC-Q-TOF/MS. Western blot was employed to detect proteins involved in the lipid metabolism-related signaling pathways. Oleic acid-induced hepatic steatosis was used to examine the lipid-lowering effect of FFDZ-containing serum *in vitro*.

**Results:**

A total of eight active metabolites were identified from the FFDZ formula and FFDZ-containing serum through UPLC-MS/MS analysis. FFDZ reduced body weight, liver weight, and levels of inflammatory cytokines, and it ameliorated hepatic steatosis, serum lipid profiles, insulin sensitivity, and glucose tolerance in mice with HFD-induced NAFLD. Transcriptomics revealed that FFDZ modulated the lipid metabolism-related pathways, including the PPAR signaling pathway, Fatty acid metabolism, and AMPK signaling pathway. Meanwhile, Western blot analysis indicated that FFDZ downregulated the expression of lipid synthesis-related proteins (Srebp-1c, Acly, Scd-1, Fasn, Acaca, and Cd36) and upregulated the fatty acid oxidation-related proteins (p-Ampk, Ppar-α, and Cpt-1). Furthermore, metabolomics identified FFDZ-mediated reversal of phospholipid dysregulation (PC, PE, LPC, LPE). Additionally, FFDZ-containing serum remarkedly reduced OA-induced lipid accumulation in HepG2 cells.

**Conclusion:**

The present results demonstrate that FFDZ exerts anti-NAFLD effects by enhancing glucose tolerance and insulin sensitivity, as well as regulating the Ampk signaling pathway to ameliorate lipid metabolism disorder, lipotoxicity, hepatic steatosis, and inflammatory responses.

## 1 Introduction

Non-alcoholic fatty liver disease (NAFLD) is one of the most common chronic liver diseases worldwide and is primarily a metabolic disease characterized by excessive accumulation of lipids in hepatocytes (Han et al., 2023; Leow et al., 2023). The spectrum of NAFLD includes simple hepatic steatosis, nonalcoholic steatohepatitis, liver fibrosis, cirrhosis, and hepatocellular carcinoma (Fraile et al., 2021). A recent meta-analysis showed that the global prevalence of NAFLD is about 30.2%, with South America exhibiting the highest prevalence at around 34% (Amini-Salehi et al., 2024). In Asia, the overall prevalence stands at approximately 30%, with China reporting a prevalence of 32.5% (Riazi et al., 2022; Teng et al., 2023). Prevalence projections indicate that by 2040, over 50% of the global adult population may be affected by NAFLD (Le et al., 2022). Unbalanced long-term energy intake results in energy metabolism disorders and is an important cause of NAFLD (Hansen et al., 2023). Disordered energy metabolism causes the accumulation of excess lipids in the liver, which can activate the sterol regulatory element binding protein-1c/fatty acid synthase/recombinant Acetyl Coenzyme A carboxylase alpha/stearoyl-CoA desaturase-1 (Srebp-1c/Fasn/Acaca/Scd-1) pathway and promote fatty acid synthesis while suppressing the fatty acid *β*-oxidation by down-regulating the expression levels of carnitine palmitoyltransferase 1 (Cpt-1), peroxisome proliferator-activated receptor α (Ppar-α), and AMP-activated protein kinase (Ampk), thereby aggravating lipid accumulation (Foretz and Viollet, 2018; Ipsen et al., 2018; Zhang et al., 2019). Furthermore, massive lipid deposition in hepatocytes causes lipotoxicity, which induces oxidative stress and promotes the formation of harmful metabolites lysophosphatidylcholine and lysophosphatidylethanolamine (LPC, LPE, etc.), which in turn triggers a series of inflammatory responses, leading to the release of inflammatory cytokines tumor necrosis factor-alpha and interleukin-6 (TNF-α and IL-6) and hepatocyte damage (Law et al., 2019; Grander et al., 2023). Additionally, insulin insensitivity is also a critical feature of NAFLD, which leads to elevated blood glucose, prompting the liver to convert excess glucose into fat, further exacerbating fat accumulation in the liver (Fang et al., 2018). Therefore, improving insulin resistance, regulating lipid metabolism, and inhibiting inflammation response are key strategies for treating NAFLD.

Traditional Chinese Medicine (TCM) has proven effective for various diseases in Chinese patients and has recently garnered interest as a potential treatment for NAFLD. Numerous studies have demonstrated that TCM can effectively treat NAFLD through the simultaneous regulation of multiple dysregulated pathways caused by massive accumulation of lipids in the liver, including the modulation of lipid metabolism, the improvement of insulin resistance, the reduction of lipotoxicity, and the attenuation of inflammatory responses (Yao et al., 2016; Xu and Cui, 2023; Wu et al., 2024). For example, Yinzhihuang granule regulates lipid metabolism in NAFLD by reducing the expression of Acaca, Fasn, and cluster of differentiation 36 (Cd36) proteins (Tan et al., 2023). Xiaozhi formula improves lipid metabolism disorders in NALFD by inhibiting the lipid synthesis pathway (Srebp-1c/Fasn) and activating the fatty acid *β*-oxidation pathway (Ppar-α/Cpt-1) (You et al., 2024). Lian-Qu formula ameliorates disease progression in mice induced with a high-fat diet (HFD) by decreasing lipogenesis, insulin insensitivity, and inflammation (Huang et al., 2023). Therefore, TCM is a promising strategy for treating NAFLD as inhibits the inflammatory response and improves insulin resistance and lipid metabolism.

Fufangduzhong formula (FFDZ) is an oral proprietary Chinese medicine with the effects of tonifying the kidney, calming the liver, and clearing heat, according to Chinese Patent Drugs enacted by the Ministry of Public Health of the People’s Republic of China (1992 Edition). Traditional Chinese medicine theory believes that liver and gallbladder dampness-heat syndrome and phlegm-dampness syndrome are the main pathogenesis of NAFLD (Zhang et al., 2020). However, according to prior research, FFDZ is often used to lower blood lipids in the treatment of hyperlipidemia and atherosclerosis in southwest minority areas of China. It consists of five botanical drugs: *Eucommia ulmoides* Oliv.*, Leonurus artemisia* (Lour.) S. Y. Hu, *Prunella vulgaris* Linn*, Uncariarhynchophylla* (Miq.) Miq. ex Havil., *and Scutellaria baicalensis* Georgi. *Eucommia ulmoides* Oliv. has many pharmacological effects, especially in treating hypertension, hyperlipemia, diabetes, and obesity (Huang et al., 2021; Zhao et al., 2022). Previous studies have reported that *L. artemisia* (Lour.) S. Y. Hu has functions related to activating blood circulation, providing detoxification, and enhancing antioxidant properties (Shang et al., 2014; Miao et al., 2019). Meanwhile, the Chinese botanical drug *P. vulgaris* Linn has been shown to be anti-inflammatory, hepatoprotective, hypoglycemic, and hypolipidemic, according to previous studies (Zhang et al., 2021; Pan et al., 2022). In addition, hirsutine, an active metabolite from *Uncariarhynchophylla* (Miq.) Miq. ex Havil., can regulate glucose homeostasis and improve insulin action (Hu et al., 2022). Also, *S. baicalensis* Georgi has been found to exert anti-inflammatory and antioxidant effects (Wang et al., 2018). Network analysis predicts that the results exhibit targets related to both NAFLD and active compounds in FFDZ, which mainly enrich the insulin resistance, AMPK signaling pathway, and PPAR signaling pathway (Supplementary Figure S1). These results suggest FFDZ plays an important role in regulating inflammation and liver metabolism and may be a potential drug for treating NAFLD. Our preliminary experiments show that FFDZ significantly improved the development of HFD-induced NAFLD mice. While the role and mechanism of FFDZ in regulating NAFLD have not been clarified.

In the present study, UPLC-MS/MS was employed to identify the potential active metabolites from FFDZ. NAFLD mice induced by HFD were used to investigate FFDZ’s activity and mechanisms against the NAFLD through serum lipid profiling, histopathological observations, liver transcriptomics, serum metabolomics, and Western blot analysis. Additionally, *in vitro* experiments were conducted to further investigate the potential mechanism of FFDZ therapy in NAFLD.

## 2 Materials and methods

### 2.1 Chemicals and reagents

Fufangduzhong formula (Z52020212) was provided by the Guizhou Dechangxiang Pharmaceutical Co., Ltd., (Guizhou, China). Oleic acid (OA), Bovine Serum Albumin (BSA), and Oil Red O (ORO) were purchased from Sigma-Aldrich (St. Louis, MO, United States). The triglyceride (TG), total cholesterol (TC), superoxide dismutase (SOD), malonaldehyde (MDA), glutathione peroxidase (GPx), aspartate aminotransferase (AST), and alanine aminotransferase (ALT) kits were purchased from Nanjing Jiancheng Bioengineering Institute (Nanjing, China). Tribromoethanol was purchased from MedChem Express (Monmouth Junction, United States, HY-B1372); Dulbecco’s modified Eagle’s medium (DMEM) was obtained from Gibco (Grand Island, NY, United States). Fetal bovine serum (FBS) from North America was obtained from VisTech (VisTech, SE100-B, South America). 4% paraformaldehyde was purchased from Servicebio Technology Co., Ltd., (Wuhan, China). Kaempferol (purity ≥98%), luteolin (purity ≥98%), quercetin (purity ≥98%), isorhamnetin (purity ≥98%), baicalein (purity ≥98%), acacetin (purity ≥98%), neobaicalein (purity ≥98%), and isorhyncophylline (purity ≥98%) were purchased from PUSH biotechnology (Sichuan, China).

### 2.2 UPLC- MS/MS analysis

The chemical composition of the FFDZ formula and serum containing the drug was analyzed by the UPLC-MS/MS method. 200 μL of FFDZ-containing serum was aspirated and mixed with 800 μL of pre-cooled methanol and left on ice for 5 min. The supernatant was collected by centrifugation at 12,000 rpm for 15 min at 4°C and dried. The supernatant was then redissolved in 50 μL of methanol and centrifuged at 12,000 rpm for 15 min to collect the supernatant for analysis. Accurately weigh 5 mg of FFDZ formula, add 1 mL of methanol solution, and ultrasonically filter to obtain 5 mg/mL of sample solution. Then, dilute this solution to prepare a 500 μg/mL solution for testing. Each standard was accurately weighed 2 mg, dissolved using methanol and mixed and diluted to a final concentration of 500 ng/mL, 250 ng/mL, 125 ng/mL, 62.5 ng/mL, 31.25 ng/mL, 15.625 ng/mL, 7.8125 ng/mL, 3.90625 ng/mL, 1.953125 ng/mL, 0.9765625 ng/mL, 0.48828125 ng/mL, 0.244140625 ng/mL and 0.1220703125 ng/mL standard solutions.

The prepared solutions were analyzed and identified on an Agilent 1290 Infinity II chromatograph (Santa Clara, CA, United States) and a Sciex Triple Quad ™ 5500+ mass spectrometer (Foster, CA, United States) using a UPLC-MS/MS system. The mobile phase A was 0.1% aqueous acetic acid, mobile phase B was acetonitrile, and the chromatographic column was Titank C18 (2.1 mm × 100 mm) with a flow rate of 0.3 mL/min at 35°C. The electrospray ionization source was used at a temperature of 500°C, and the spray voltage was 5.5 kV for positive ion mode and 4.5 kV for negative ion mode. The ion source gas 1 was 50, the auxiliary pressure was 50, and the collision energy was 34 eV; multiple reaction detection (MRM) modes.

### 2.3 Animal experiments

The procedure for the animal experiment was approved by the accredited animal ethics committee of Guizhou Medical University and the Council of Animal Care (Approval No. #SYXKQIAN-2018-0001, July 12, 2022). C57BL/6J mice (6 weeks old) were purchased from the Model Animal Research Center of Nanjing University (Nanjing, China). The mice were housed in an SPF environment and kept at a temperature of 20°C-25°C, with a humidity of 60% and a cycle of 12 h of light and 12 h of darkness to ensure that the mice were free to drink and eat. After 1 week of acclimatization feeding, the mice were randomly divided into four groups, control group (ND), high-fat diet group (HFD), FFDZ low-dose group (0.45 g/kg/d) (HFD+L), and FFDZ high-dose group (0.9 g/kg/d) (HFD+H). The ND mice were fed with normal chow, and the HFD, HFD+L, and HFD+H groups were fed with high-fat chow (60% energy from fat, D12492, Research diet, New Brunswick, United States). The HFD (D12492, Research Diets) is a well-established model for inducing obesity, hyperlipidemia, and NAFLD in rodents, with 60% fat-derived calories, as validated in prior studies (Clifford et al., 2021; Huang et al., 2024). The mice were given the corresponding daily dose of the drug by gavage, and the drug was replaced by saline in ND and HFD groups for 12 weeks, and the mice were weighed every week. In the last week, the fasting blood glucose (FBG) levels and the oral glucose tolerance test (OGTT) were detected using an Accu-Chek Active Blood Glucose Meter (Roche). The integrated area under the curve (AUC) was used to analyze glucose tolerance in all groups.

### 2.4 Biochemical analysis

Blood was taken from the fundus venous plexus of mice after carbon dioxide inhalation anesthesia, and mouse serum was collected by centrifugation at 4,000 rpm for 10 min at 4°C. Serum levels of AST, ALT, TG, TC, HDL-C, LDL-C, SOD, GPx, and MDA in different groups of mice were measured using an automated biochemical analyzer. The levels of IL-6, TNF-α, and insulin (INS) were detected in mice serum using ELISA kits according to the instructions (Nanjingjiancheng, Nanjing, China) (Zhang et al., 2023; Zhu et al., 2023; Huang et al., 2024). Homeostasis model assessment insulin resistance (HOMA-IR) was calculated as follows: HOMA-IR = FBG × FINS/22.5.

### 2.5 Histological analysis

Histological analysis was performed using Oil red O (ORO) staining and hematoxylin-eosin (H&E) staining. At the end of blood sampling from the fundus venous, mice were euthanized from the cervical dislocation to remove mouse liver tissue and weighed. For H&E staining, liver tissues were fixed with a 4% paraformaldehyde fixative solution, and the tissues were dehydrated, embedded in paraffin, and sectioned. For ORO staining, the sections were stained with ORO, toned with 60% isopropyl alcohol, and then stained with hematoxylin. All stained sectioned tissues were photographed with a microscope.

### 2.6 Liver transcriptome sequencing

RNA from liver tissue was extracted according to the TRIzol reagent’s instructions (Invitrogen, United States). The concentration of RNA was measured using a NanoDrop 2000 (Thermo Fisher Scientific, Wilmington, DE), and the quality of RNA was controlled using Agilent Bioanalyzer 2100. Library construction and sequencing of RNA were performed at Beijing Genomics Institute (BGI, Wuhan, China) using the DNBSEQ platform. The raw data obtained from sequencing were filtered using SOAPnuke. The filtered data, referred to as clean data, were subsequently analyzed, visualized, and mined using Dr. Tom online analysis platform. The reference genome was compared using HISAT software, and differential gene detection (Q value ≤ 0.05 or FDR ≤ 0.001) was performed using DESeq2 GO and KEGG enrichment analyses of differential genes were performed using Phyper, and Q values ≤ 0.05 were significantly enriched in the candidate genes. The results were visualized using TBtool software.

### 2.7 Metabolomics analysis

The 100 μL of serum samples were mixed with 400 μL of pre-cooled methanol/acetonitrile (1:1, v/v) and allowed to stand on an ice bath for 5 min, and the supernatant was collected by centrifugation at 12,000 rpm for 15 min at 4°C and dried. The samples were redissolved in 100 μL acetonitrile/water (1:1, v/v) before analyzing each group of samples using UPLC-Q-TOF/MS. The processed data were subjected to multivariate statistical analyses, including Pareto-scaled principal component analysis (PCA) and orthogonal partial least squares-discriminant analysis (OPLS-DA), by the R package (ropls). 7-fold cross-validation and response replacement tests were used to assess the robustness of the model. Metabolites with significant changes were screened based on Variable importance of projection (VIP) > 1 and P value < 0.05.

### 2.8 Western blot

Mouse liver tissues were lysed using RIPA lysis solution containing 1% PMSF and 1% phosphatase inhibitor. Then, the liver tissues were ground into homogenate using a tissue grinder and placed on ice for 40 min. The supernatant was obtained after centrifuged at 12,000 rpm for 15 min. The protein content was detected using the bicinchoninic acid assay (BCA) protein concentration assay kit. The extracted proteins were isolated by sodium dodecyl sulfate-polyacrylamide gel electrophoresis (SDS-PAGE), transmembrane and closed for 2 h using 5% skimmed milk or 3% BSA, and incubated overnight at 4°C with primary antibodies including β-actin (AF7018, Affinity), Cpt-1 (ab234111, Abcam), Ppar-α (ab178865, Abcam), Cd36 (ab133625, Abcam), Ampkα (AF6423, Affinity), p-Ampk (AF3423, Affinity), Acaca (ab45174, Abcam), stearoyl-CoA desaturase-1([SCD-1] #2438, CST), SREBP-1c (AF6283, Affinity), Fasn (DF6106, Affinity), and ATP citrate lyase ([Acly] AF7832, Affinity). The secondary antibodies were incubated at room temperature for 1 h and then developed using an Odyssey imager to save the images.

### 2.9 FFDZ-containing serum preparation

SD rats of 6–8 weeks (200 ± 20 g) with certificate number SCXK (Jing) 2019-0008, were supplied by Beijing Huafukang Biotechnology Corporation Limited (Beijing, China). After 1 week of acclimatization feeding, they were prepared and randomly divided into two groups, normal (Control) and FFDZ formula (FFDZ). The drug was given to the rats by gavage once a day for five consecutive days. The rats were fasted but not watered on the night of the fourth day and anesthetized by intraperitoneal injection of tribromoethanol (0.3 mL/100 g) 2 h after drug administration on the morning of the fifth day. After anesthesia, blood was taken via the abdominal aorta, left at 4°C for 2 h, and then centrifuged at 3,000 rpm for 15 min to collect the upper serum layer. The collected serum was inactivated in a water bath at 56°C for 30 min, filtered and partitioned, and stored in a refrigerator at −80°C.

### 2.10 Analysis of lipids in HepG2 cells

Human hepatocellular carcinoma cells (HepG2) were purchased from the Chinese Academy of Sciences (Shanghai, China). HepG2 cells were cultured using Dulbecco’s modified Eagle’s medium (DMEM) medium containing 10% FBS, and incubated at 37°C with 5% CO_2_ in an incubator until the cell confluence reached approximately 90%. HepG2 cells in the logarithmic growth phase were inoculated into 6-well plates at a density of 1.5 × 10^5^ cells per well. After 12 h of wall affixation, the cells in the 6-well plates were treated with 0.5 mM oleic acid-bovine serum albumin (OA-BSA) complex for 24 h. The OA-containing medium was removed, and the cells in 6-well plates were washed with PBS before adding fresh serum-free DMEM medium and incubated for 48 h under the condition of containing FFDZ-containing serum (2.5%, 5%, and 10%) or normal mice serum. TG levels were detected according to the instructions of the TG kit. Oil Red O staining in cells was performed according to our previous study (Lei et al., 2023).

### 2.11 Statistical analysis

The experimental data were statistically analyzed using SPSS 26.0 software, and the results were expressed as mean ± standard deviation. Comparisons between multiple groups were statistically analyzed using One-way analysis of variance (ANOVA) and were considered statistically significant at *p* < 0.05. Graphs were generated using GraphPad Prism 8.0.2.

## 3 Results

### 3.1 FFDZ composition analysis

**FIGURE 1:**
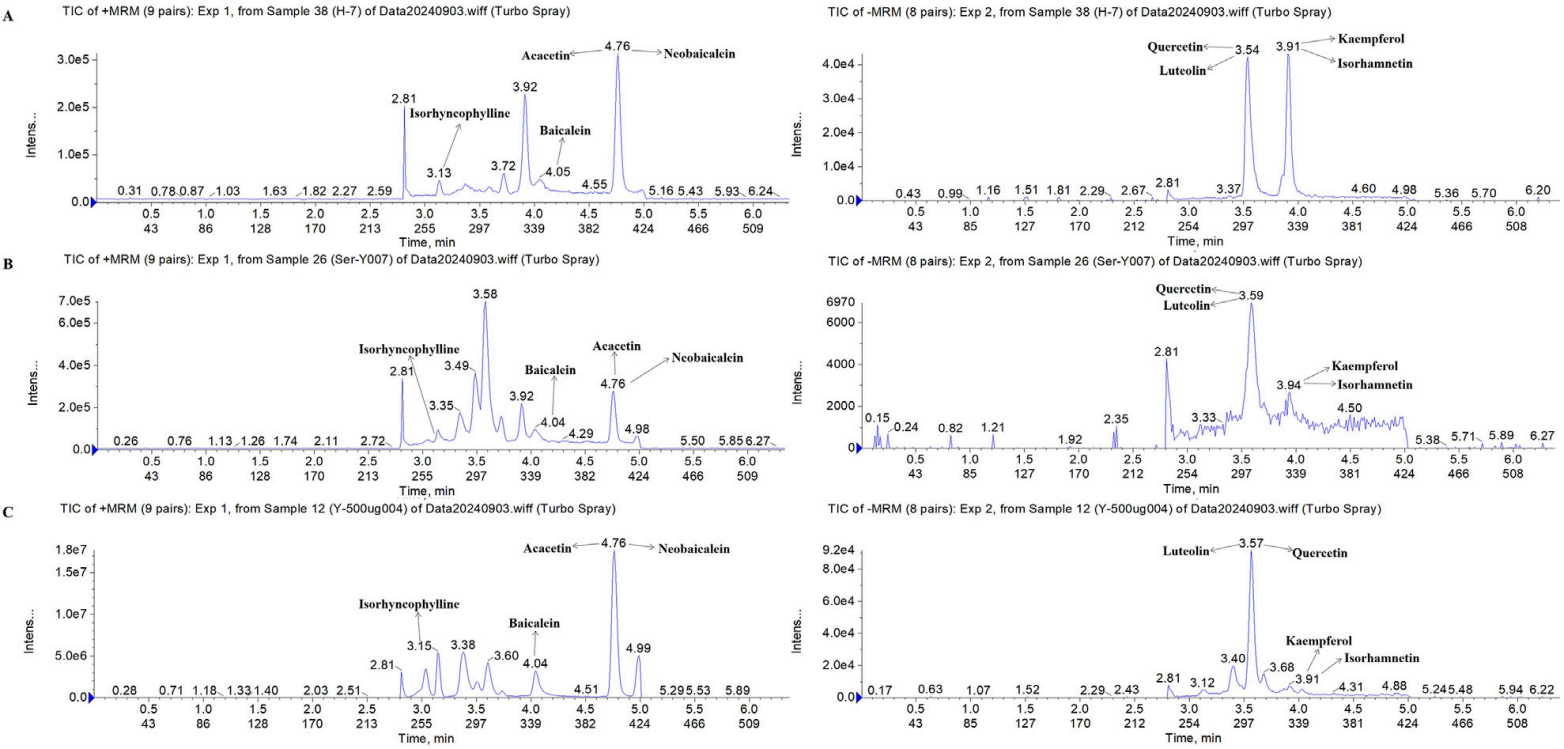
Compositional analysis of FFDZ by UPLC-MS/MS. **(A)** 8 mixed standards; **(B)** FFDZ-containing serum; **(C)** FFDZ formula solution.

To identify the chemical metabolites of FFDZ and their intestinal absorption, a UPLC-MS/MS method was established. As a result, eight metabolites were identified from the FFDZ formula and drug-containing serum based on UPLC-MS/MS results, including Kaempferol, Luteolin, Quercetin, and Isorhamnetin in a negative ion mode, as well as Baicalein, Acacetin, Neobaicalein, and Isorhyncophylline in positive ion mode. The base peak intensity (BPI) chromatograms of FFDZ and FFDZ-containing rat serum in negative and positive ion modes are shown in Figure 1. Their associated retention times and concentrations are shown in Table 1.

**TABLE 1:** UPLC-MS/MS characterized the chemical composition of the FFDZ formula and FFDZ-containing serum samples.

No.	Metabolite name	Formula	RT	Ion mode	m/z	FFDZ formula (ng/mL)	Serum (ng/mL)
1	Kaempferol	C15H10O6	3.87	ESI−	285.10	111.83	6.42
2	Luteolin	C15H10O6	3.56	ESI−	285.10	6.14	0.11
3	Quercetin	C15H10O7	3.59	ESI−	301.10	52.71	7.22
4	Isorhamnetin	C16H12O7	3.93	ESI−	315.00	1.87	0.64
5	Baicalein	C15H10O5	4.06	ESI+	271.10	6432.87	279.47
6	Acacetin	C16H12O5	4.78	ESI+	285.20	91.07	1.92
7	Neobaicalein	C19H18O8	4.79	ESI+	375.20	266.68	2.67
8	Isorhyncophylline	C22H28N2O4	3.10	ESI+	385.30	58.33	0.19

### 3.2 FFDZ improves body weight and lipid accumulation in HFD-induced NAFLD mice

**FIGURE 2:**
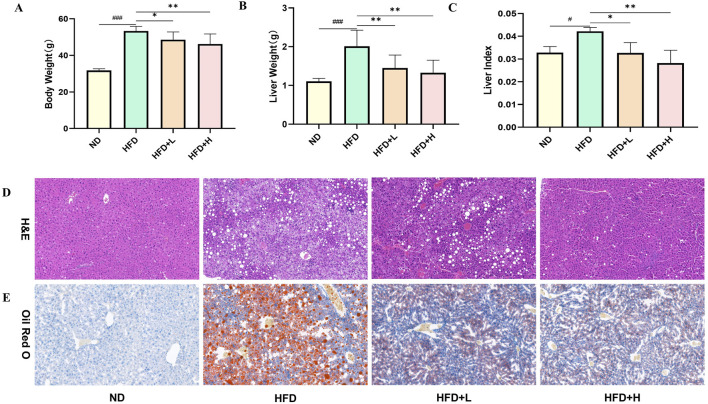
Effect of FFDZ on HFD-induced NAFLD mice. **(A)** Body weight; **(B)** Liver weight; **(C)** Liver index; **(D)** H&E staining (×200 magnification); **(E)** ORO staining (×400 magnification). Data are means ± standard deviations (SD, n = 7). ^###^ *P* < 0.001 compared with the ND group; *P < 0.05 and **P < 0.01 compared with the HFD group.

In order to investigate the therapeutic effect of FFDZ on NAFLD, C57BL/6J mice were administered HFD with or without FFDZ for 12 weeks. As outlined in Figure 2A, the body weight of mice in the HFD group (53.74 ± 2.60 g) was significantly higher than that in the ND group (31.79 ± 0.89 g) induced by the HFD. However, after 12 weeks of continuous administration of FFDZ, the increasing tendency was significantly reversed with the increase of FFDZ compared to the HFD group, especially in the HFD+H group (45.30 ± 5.45 g). Consistent changes in liver weight and liver index were markedly increased by 81.7% and 28.6%, respectively, in the HFD group compared with the ND group, while the FFDZ intervention reversed these changes remarkably (Figures 2B, C). In addition, histopathological observations revealed ballooning degeneration and excessive lipid droplet accumulation in the livers of mice with HFD-induced NAFLD (Figures 2D, E), suggesting that the HFD caused hepatic steatosis. Notably, liver sections in the HFD-induced NFLD mice showed very mild hepatic steatosis following the administration of FFDZ, particularly in the high-dose group (Figures 2D, E). These results indicate that the FFDZ effectively attenuated hepatic steatosis in mice with NAFLD caused by HFD.

### 3.3 FFDZ improves serum biochemical indices in NAFLD mice

**FIGURE 3:**
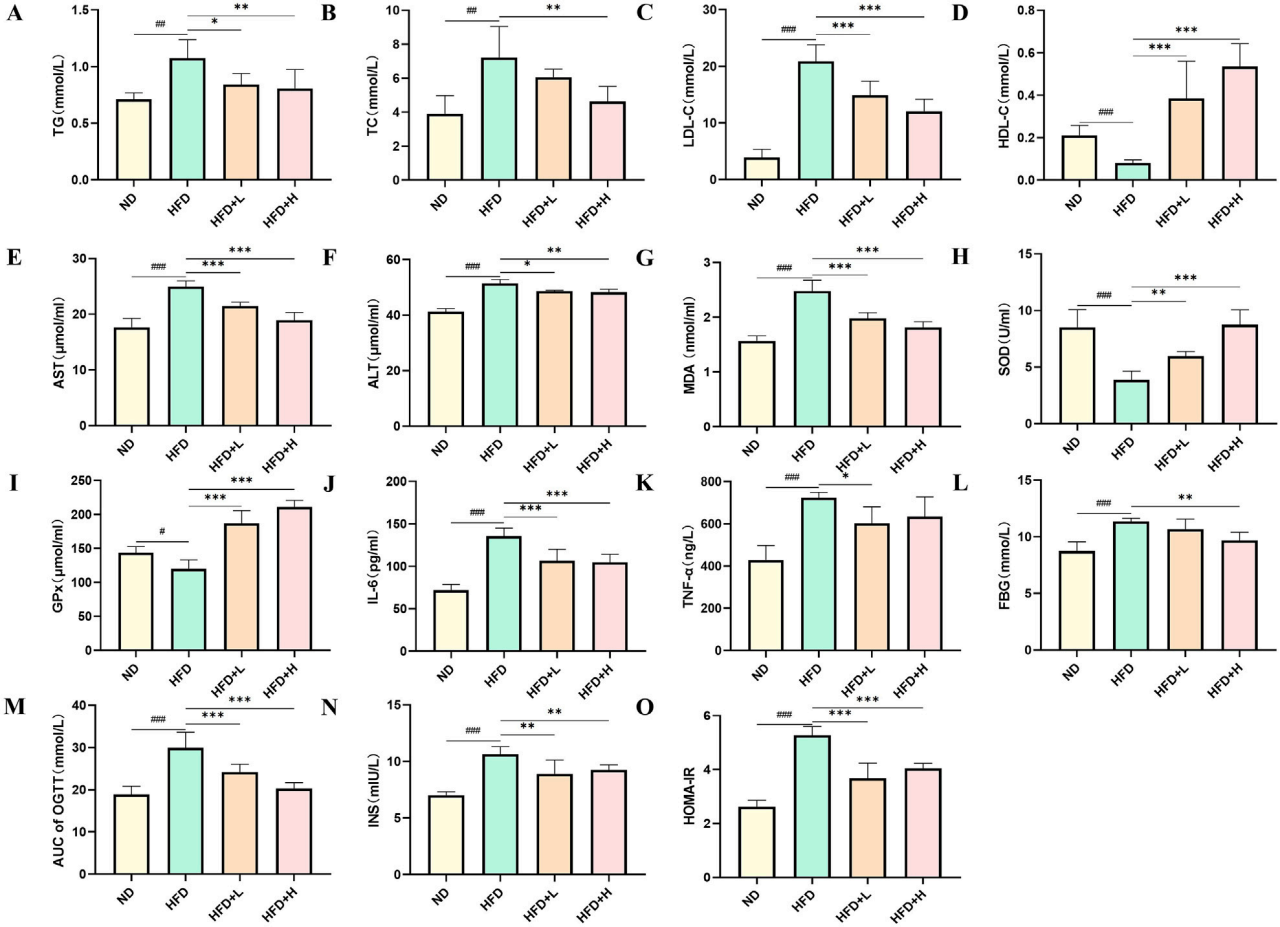
Effect of FFDZ on serum biochemical indices in HFD-induced NAFLD mice. **(A)** TG; **(B)** TC; **(C)** LDL-C; **(D)** HDL-C; **(E)** AST; **(F)** ALT; **(G)** MDA; **(H)** SOD; **(I)** GPx; **(J)** IL-6; **(K)** TNF-α; **(L)** FBG; **(M)** AUC of OGTT; **(N)** INS; **(O)** HOMA-IR. Data are means ± standard deviations (SD, n = 7). ^#^ *P* < 0.05, ^##^ *P* < 0.01 and ^###^ *P* < 0.001 compared with the ND group; *P < 0.05, **P < 0.01 and ***P < 0.001 compared with the HFD group.

To investigate the effects of FFDZ on lipid homeostasis, we measured the serum lipid profiles in mice. As shown in Figures 3A–D, serum TG, TC, and LDL-C levels in the HFD-induced mice were remarkedly elevated by 0.5-, 2.3-, and 4.4-fold, respectively, in comparison to the ND group, while serum HDL-C levels were decreased by 0.6-fold. On the contrary, FFDZ administration remarkably decreased serum TG, TC, and LDL-C levels in the HFD-fed mice in a dose-dependent way, whereas changes in serum HDL-C levels showed a gradual downward trend. Furthermore, lipotoxicity induced by massive lipid deposition in the liver resulted in serum AST, ALT, and MDA levels in HFD-induced mice that were 41.3%, 24.6%, and 58.15% higher, respectively, than those in the ND group, while FFDZ intervention dose-dependently reversed these changes (Figures 3E–G). Of interest, serum SOD and GPx levels were decreased by 54.42% and 16.48% in the HFD-fed mice, respectively, compared with the ND group, as outlined in Figures 3H, I. Following treatment with FFDZ, serum SOD and GPx levels exhibited an increasing trend with higher doses of the drug, particularly in the high-dose FFDZ group, where the levels were comparable to those in the NC group. Additionally, HFD remarkedly triggered the inflammatory responses, leading to increases in serum IL-6 and TNF-α levels in the HFD-induced mice by 88.24% and 69.05%, respectively, compared with the ND group, whereas those levels exhibited a decreasing trend as the FFDZ dose increased (Figures 3J, K). These data suggest that FFDZ improved the lipid profile and inhibited lipotoxicity and inflammatory responses caused by a disorder of serum lipid homeostasis in mice with HFD-induced NALFD.

NAFLD is a metabolic disorder characterized by glucose intolerance and insulin resistance, resulting in the aggravation of lipid metabolism disorders and lipid accumulation in the liver (Mota et al., 2016). In this study, we examined the glucose levels of mice in each experimental group in the 12th week following FFDZ administration. As presented in Figure 3L, FFDZ treatment dose-dependently reversed the increase in fasting blood glucose (FBG) levels in NAFLD mice caused by a HFD. Furthermore, we measured glucose tolerance in HFD-induced NAFLD mice via oral glucose tolerance test (OGTT) following administration with FFDZ. Consist with changes in FBG, FFDZ administration significantly decreased the AUC of OGTT in the HFD-induced mice in a dose-dependent pattern; notably, the result in the high-dose FFDZ group was similar to that of the ND group (Figure 3M). Also, HFD feeding caused a significant increase in serum insulin levels in the HFD group of mice, with an increase of 51.8% compared to the ND group. In contrast, FFDZ intervention significantly reduced the serum insulin level in the HFD-fed NAFLD mice, particularly in the high-dose group (Figure 3N). Meanwhile, following treatment with FFDZ, the changes in HOMA-IR in the NAFLD mice exhibited a trend like that of serum insulin and glucose levels, as shown in Figure 3O. These results indicate that FFDZ enhanced the insulin sensitivity and glucose tolerance in the HFD-fed NAFLD mice.

### 3.4 FFDZ regulates metabolic levels in NAFLD mice

**FIGURE 4:**
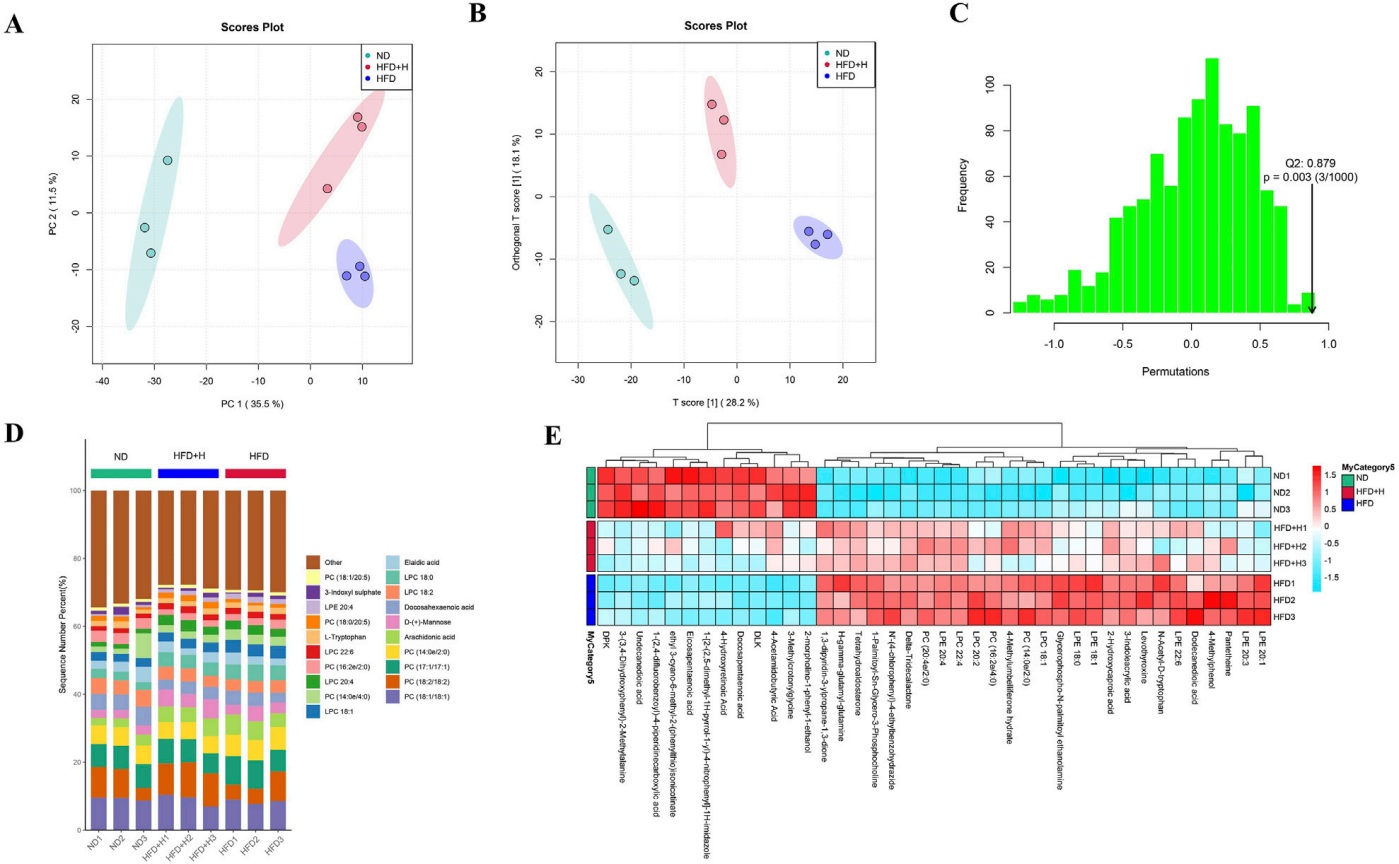
Serum untargeted metabolomics analysis of NAFLD mice. **(A)** PCA score plots; **(B)** OPLS-DA score plots; **(C)** Model validation plot for OPLS-DA; **(D)** Metabolite stacking diagram; **(E)** Metabolite clustering heat map. Data are means ± standard deviations (SD, n = 3).

To further analyze the effect of FFDZ on NAFLD, we used untargeted metabolomics to detect changes in metabolite levels in serum. Serum samples from each experimental group were analyzed using UPLC-Q-TOF/MS, and the data were subjected to PCA and OPLS-DA with model validation. As shown in Figures 4A–C, the PCA and OPLS-DA plots of the HFD group and the other groups showed significant separation, indicating that the groups showed good variability, and Q2 > 0.8 in the simulation validation plots also proved the reliability of the developed model. In the OPLS-DA model, we considered the differential metabolites with VIP > 1.0 and *p* < 0.05 to be significant, and the HFD group had 696 different metabolites compared with the ND group, while the FFDZ group reversed the expression of 422 of these metabolites. Compared to the ND group, the serum levels of PC (20:4e/2:0, 16:2e/4:0, 14:0e/2:0), LPC (22:4, 22:6, 20:2, 18:1, 18:0), LPE (22:6, 20:4, 20:1, 20:3, 18:1, 18:0), and arachidonic acid in the HFD group were significantly increased, as outlined in Figures 4D, E. In contrast, the content of PC (18:2/18:2, 18:1/20:5, 18:0/20:5, 18:1/18:1) in the HFD group was markedly decreased. However, the high dose of FFDZ intervention significantly reversed these changes (Supplementary Table S1). The above result indicates that FFDZ can improve the metabolic disorders induced by HFD in NAFLD mice.

### 3.5 FFDZ affected the gene expression in the liver of NAFLD mice

**FIGURE 5:**
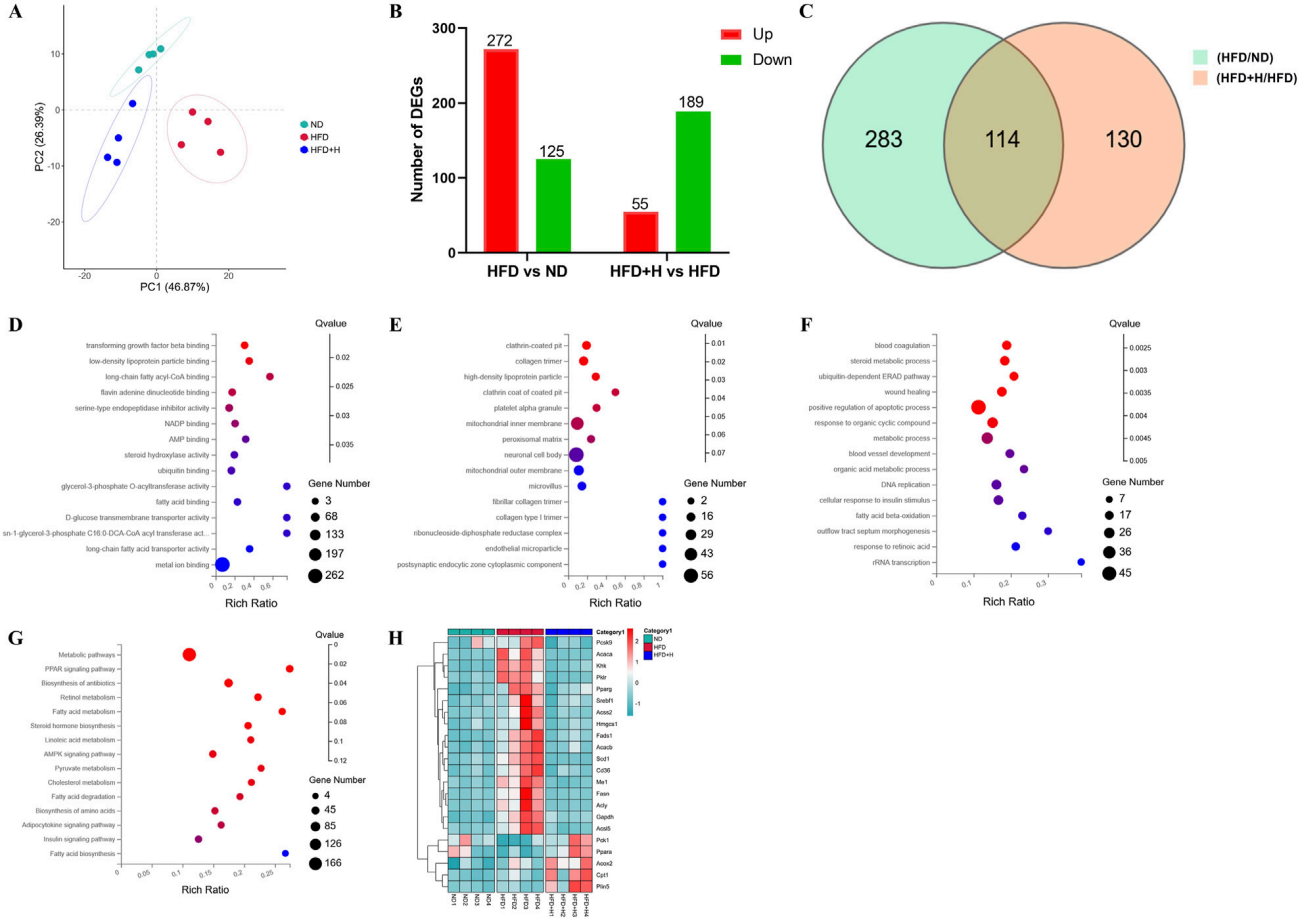
Transcriptomics analysis of the liver on FFDZ treatment. **(A)** PCA; **(B)** Differentially expressed genes (DEGs) between HFD and ND group, as well as HFD+L and HFD group; **(C)** Venn diagrams; **(D)** Molecular functions (MF); **(E)** Cellular components (CC); **(F)** Biological process (BP); **(G)** KEGG; **(H)** heat map. Data are means ± standard deviations (SD, n = 3).

To investigate the fundamental mechanism by which FFDZ intervention exerts an improvement of hepatic steatosis in NAFLD mice, we analyzed the hepatic gene expression profile by using RNA-seq. As shown in Figure 5A, the PCA plots showed a clear separation of the ND, HFD, and HFD+H groups, while the HFD+H group was much closer to the ND group than the HFD group alongside PC1, suggesting the HFD+H group has a higher similarity to the ND group in hepatic gene expression, rather than to the HFD group. Next, the differentially expressed genes (DEGs) were filtered by the P adjust value (*P* < 0.05) and fold change (FC > 1.2), including between HFD and ND groups as well as HFD+H and HFD groups were shown in Figures 5B, C. We found 397 DEGs between the HFD and ND group and 244 DEGs between the HFD+H and HFD group.

Furthermore, we described the primary function of common DEGs by using GO and KEGG enrichment analysis. The GO enrichment showed that the main molecular functions (MF) included low-density lipoprotein particle binding, long-chain fatty acyl-CoA binding, AMP binding, fatty acid binding, etc. For cellular components (CC), the DEGs were significantly enriched in high-density lipoprotein particles, inner membrane, peroxisomal matrix, etc. The main biological process (BP) included the steroid metabolic process, fatty acid beta-oxidation, metabolic process, and cellular response to insulin stimulus (Figures 5D–F). And KEGG pathway enrichment analysis results exhibited that the function of DEGs focused on the PPAR signaling pathway, fatty acid metabolism, AMPK signaling pathway, insulin signaling pathway, and fatty acid degradation (Figure 5G), all of which were closely related to the progression of NAFLD. It is similar to the predicted results found in the network analyses of FFDZ (Supplementary Material). Additionally, a clustered heat map analysis displayed that lipid metabolism-related DEGs, which played a key role in maintaining lipid homeostasis in the body, were disrupted by HFD, whereas those were reversed by the FFDZ administration significantly (Figure 5H). The above results indicate that FFDZ can improve the abnormal hepatic gene expression profile involved in lipid metabolism in mice caused by HFD.

### 3.6 FFDZ improves hepatic steatosis of HFD-induced NAFLD by altering the levels of lipid metabolism-related proteins

**FIGURE 6:**
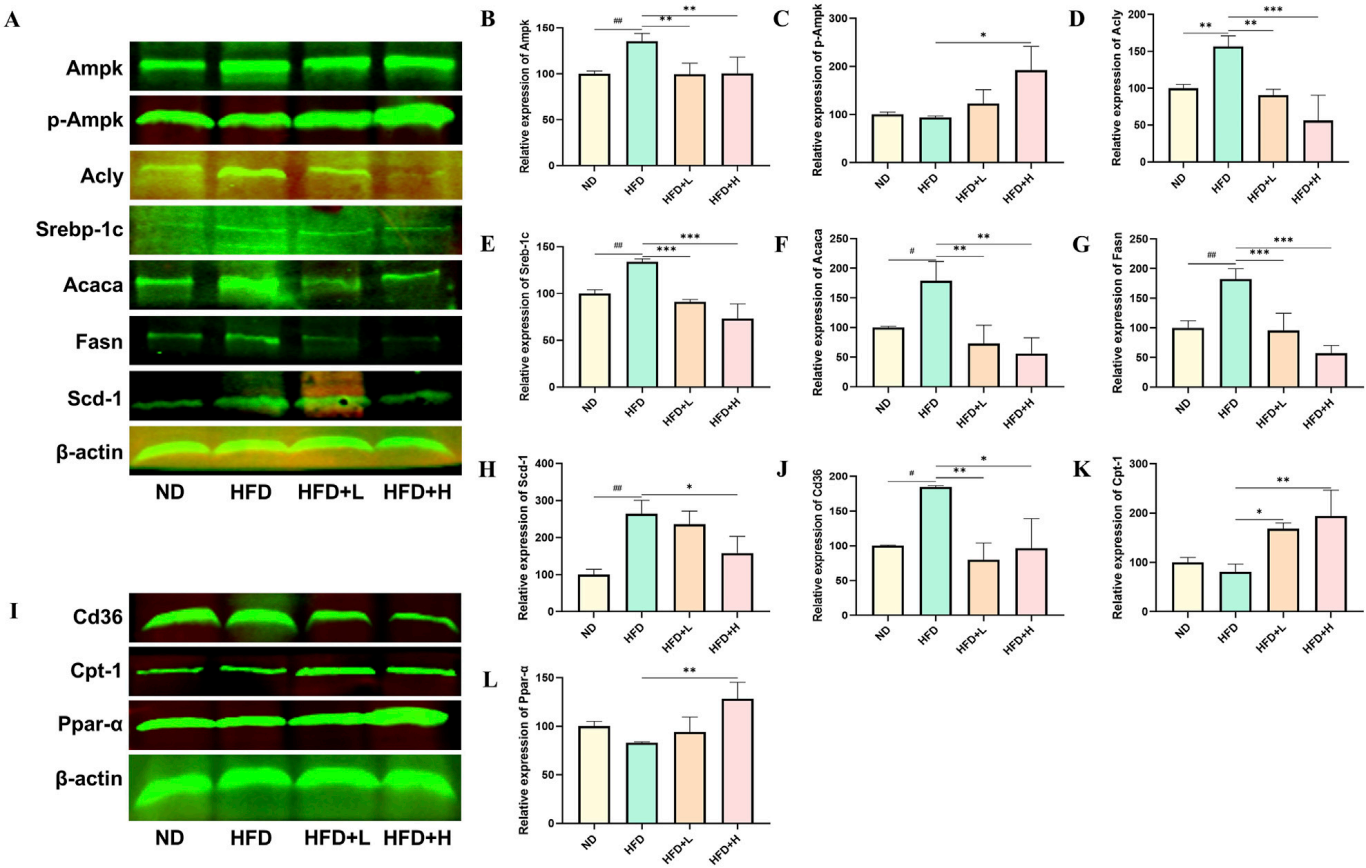
Effect of FFDZ on lipid synthesis and fatty acid oxidation-related proteins in HFD-induced NAFLD mice. **(A–H)** Statistical graph of expression levels of Ampk, p-Ampk, Acly, Srebp-1c, Acaca, Fasn, Scd-1, and β-actin related proteins; **(I–L)** Statistical graph of expression levels of Cd36, Cpt-1, Ppar-α, and β-actin related proteins. Data are means ± standard deviations (SD, n = 3). ^#^ *P* < 0.05 and ^##^ *P* < 0.01 compared with the ND group; *P < 0.05, **P < 0.01 and ***P < 0.001 compared with the HFD group.

To further explore the molecular mechanisms by which FFDZ improves NAFLD, we examined the expression levels of proteins related to lipid synthesis and fatty acid oxidation in liver tissues, based on the results of transcriptomics and serum lipid profile. As depicted in Figures 6A–H, compared to the ND group, the expression levels of Srebp-1c, Acly, Scd-1, Fasn, and Acaca, which play a crucial role in lipid synthesis, were increased in the HFD group by 33.97%, 56.44%, 164.52%, 82.53%, and 57.89%, respectively. However, those expressions of levels in the liver of NAFLD mice were dose-dependently reversed following treatment with FFDZ, compared with the HFD group. Of interest, the Ampk protein expression level in the HFD group of mice was 35.24% higher than that in the ND group, while FFDZ significantly reversed this trend. Nonetheless, the change in the trend of Ampk was opposite to that of p-Ampk. Furthermore, HFD feeding significantly decreased the expression levels of fatty oxidation-related proteins Ppar-α and Cpt-1, whereas their expression increased as the dose of FFDZ increased, showing upregulation by 0.6- and 1.0-fold in the high-dose FFDZ group compared to the HFD group (Figures 6I, K, L). In addition, Cd36, as a key protein for fatty acid transport, was significantly upregulated by 84.67% in the HFD group compared with the ND group, while FFDZ markedly reversed the trend in a dose-dependent way (Figures 6I, J). These results indicate that FFDZ may treat NAFLD by inhibiting the expression of lipid synthesis-related protein levels and up-regulating the lipid oxidation-related protein expression.

### 3.7 Correlation analysis of biochemical indices, proteins, and differential metabolites

**FIGURE 7:**
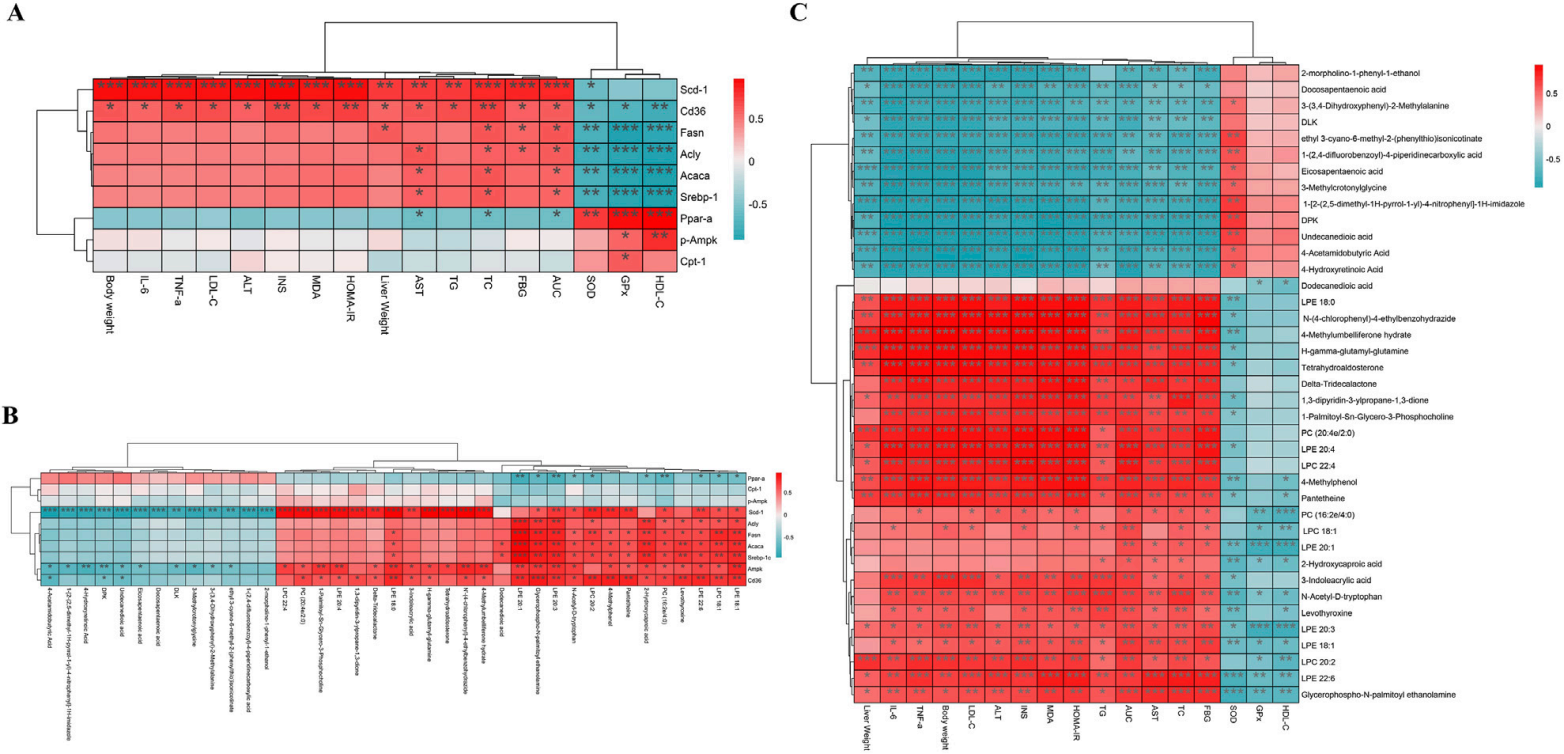
Spearman’s correlation analysis. **(A)** The relationship between biochemical indicators and protein; **(B)** The relationship between protein and differential metabolites; **(C)** The relationship between biochemical indicators and differential metabolites. Red represents positive correlation and green represents negative correlation. *P < 0.05, **P < 0.01 and ***P < 0.001.

To investigate the relationship between FFDZ efficacy and its mechanism of action, we performed Spearman’s correlation analysis to explore the connections among physiological and biochemical indicators, lipid metabolism-related proteins, and differential metabolites. Specifically, we examined the relationships between physiological and biochemical indicators and lipid metabolism-related proteins, differential metabolites and lipid metabolism-related proteins, and physiological and biochemical indicators and differential metabolites. As shown in Figure 7A, the body weight, liver weight, and serum biochemical indices (IL-6, TNF-α, TG, TC, LDL-C, AST, ALT, MDA, INS, HOMA-IR, FBG, and AUC) were positively correlated with lipid synthesis-related proteins, but negatively correlated with oxidation-related proteins, including Ppar-α and Cpt-1. Notably, the SOD, GPx, and HDL-C had a significant negative correlation with Ampk, Srebp-1c, Acly, Fasn, Acaca, and Cd36, whereas a positive correlation was seen with p-Ampk, Ppar-α, and Cpt-1. Furthermore, from the heatmap (Figure 7B), we found that lipid synthesis-related were positively correlated with differential metabolites, such as PC (20:4e/2:0, 16:2e/4:0), LPC (22:4, 20:2, 18:1), and LPE (22:6, 20:4, 20:1, 20:3, 18:1, 18:0). In contrast, p-Ampk, Ppar-α and Cpt-1 were negatively correlated with PC (16:2e/4:0), LPC (20:2, 18:1), and LPE (22:6, 20:1, 20:3, 18:1). In addition, differential metabolites also exhibited close relationship with physiological and biochemical indicators. Figure 7C showed that PC (20:4e/2:0, 16:2e/4:0), LPC (22:4, 20:2, 18:1), and LPE (22:6, 20:4, 20:1, 20:3, 18:1, 18:0) were positively correlated with body weight, liver weight, IL-6, TNF-α, TG, TC, LDL-C, AST, ALT, MDA, INS, HOMA-IR, FBG, and AUC, while negatively correlated with SOD, GPx and HDL-C.

### 3.8 FFDZ-containing serum ameliorates OA-induced lipid accumulation in HepG2 cells

**FIGURE 8:**
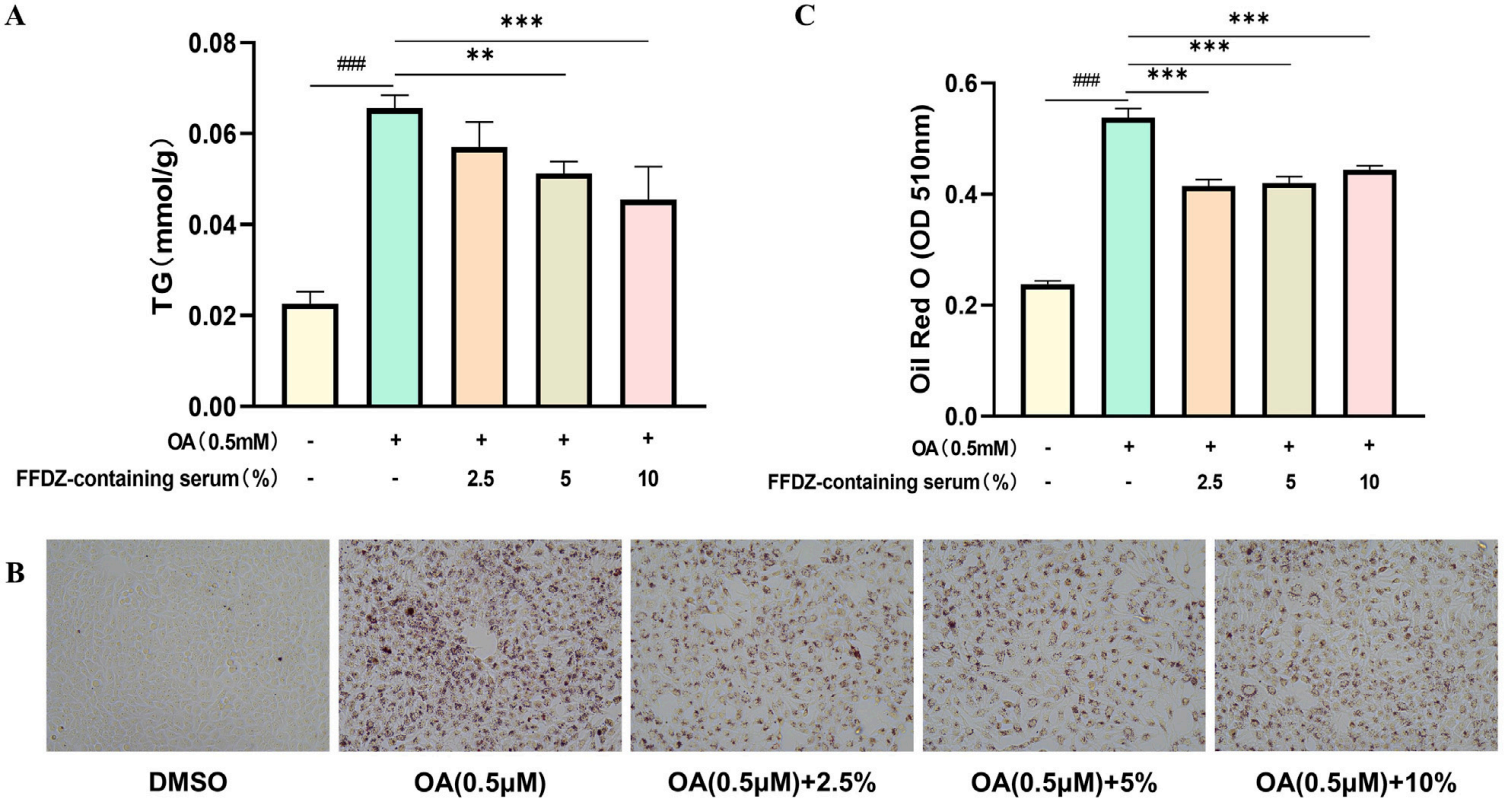
Effect of FFDZ containing serum on OA-induced hepatic steatosis model in HepG2 cells. **(A)** TG levels in cells; **(B)** Observation of cellular lipid accumulation after ORO staining under the light microscope (×200 magnification); **(C)** Mean value of ORO staining in cells at 510 nm. Data are means ± standard deviations (SD, n = 3). ^###^ *P* < 0.001 compared with the control group; **P < 0.01 and ***P < 0.001 compared with the OA group.

To further explore the effect of FFDZ on the dysregulation of lipid metabolism, we assessed the lipid-lowering efficacy of FFDZ-containing serum in improving a hepatic steatosis model caused by oleic acid (OA) in HepG2 cells. As presented in Figure 8A, the OA intervention substantially increased TG levels in the OA-induced HepG2 cells by 2.9-fold compared to the normal group, while the FFDZ-containing serum reduced cellular TG production caused by OA in a concentration-dependent manner. Furthermore, consistent with the change in TG levels, the deposition of lipid droplets in HepG2 cells induced by OA was substantially reduced with increasing concentrations of FFDZ-containing serum, as indicated by ORO staining, compared to the OA-induced group, as depicted in Figures 8B, C. These results indicate that FFDZ-containing serum can effectually reduce the deposition of lipid droplets in hepatocytes caused by OA.

## 4 Discussion

NAFLD has become one of the most prevalent chronic liver diseases worldwide and is a major cause of liver-related morbidity and mortality (Byrne and Targher, 2015). Currently, a long-term high-fat and high-sugar diet, along with an imbalance in energy expenditure, further promotes lipid metabolism disorders, which are the leading causes of NAFLD (Softic et al., 2016). Previous studies indicate that lipid metabolism disorders cause abnormalities in multiple signaling pathways in NAFLD patients, such as insulin resistance, glucose intolerance, inflammatory responses, and a decrease in antioxidant defense function (Buzzetti et al., 2016; Chen et al., 2020), thereby making it one of the most challenging diseases to prevent and treat in the world. Currently, although there are many studies on NAFLD, there is no satisfactory treatment strategy for NAFLD yet. Therefore, there is an urgent need to find new multi-target drugs to treat NAFLD. Our preliminary experimental study found that the TCM FFDZ can significantly ameliorate the progression of NAFLD mice induced by HFD, and thus it may have potential effects in treating NAFLD due to its multi-component, multi-target, and multi-pathways properties. However, further research needs to be performed to clarify the role of FFDZ in the treatment of NAFLD.

In the present study, we conducted the NAFLD mice, which were induced by feeding with HFD for 12 weeks, to investigate the effect of FFDZ. We examined the abnormal changes in physiological and biochemical indicators, including body weight, hepatic steatosis, insulin sensitivity, glucose metabolism, serum lipid profile, inflammatory response, and antioxidant defense function, which are key indicators of NAFLD development (Cobbina and Akhlaghi, 2017; Bessone et al., 2019). Results revealed that FFDZ significantly decreased the body and liver weight, reduced lipid droplet deposition, and improved hepatic steatosis in the mice with HFD-induced NAFLD. Consistent with this result, the FFDZ-containing serum also decreased the lipid accumulation in OA-induced HepG2 cells. Like physiological indicators changes, FFDZ improved and reduced the serum TG, TC, and LDL-C levels, while increasing the HDL-C level. Furthermore, previous studies found that HFD also causes glucose metabolism disorder and insulin insensitivity, which are important driving factors in the occurrence of NAFLD and NASH (Lian et al., 2020), whereas FFDZ intervention markedly enhanced insulin sensitivity and glucose tolerance. These results indicated that FFDZ significantly improved the development of HFD-fed NAFLD mice by attenuating lipid and glucose metabolism disorders and enhancing insulin sensitivity. In addition, FFDZ administration significantly reversed the increase in inflammatory cytokines (IL-6 and TNF-α) in HFD-induced NAFLD mice, which are key markers of NAFLD progression to NASH (Duan et al., 2022). This result demonstrates that FFDZ can inhibit the inflammatory response in NAFLD mice induced by lipid metabolism disorders. Noteworthy, FFDZ dramatically increased serum SOD and GPx levels while reducing liver function indices and MDA levels in NAFLD mice, thereby repairing the liver damage induced by lipid metabolism disorders, which is consistent with previous research findings (Huang et al., 2024). These findings indicate that FFDZ can facilitate the progression of mice with NAFLD induced by HFD. Therefore, the underlying mechanism of FFDZ requires further study and may offer potential new therapeutic strategies for the clinical management of NAFLD.

Next, we used UPLC-Q-TOF/MS to analyze the differential changes in metabolic substances in the serum of HFD-induced NAFLD mice, as changes in the levels of lipid metabolites can exacerbate hepatocyte damage and contribute to systemic metabolic dysfunction (Lai et al., 2015; Wang et al., 2022). In this study, results displayed 39 potential biomarkers, including PC (20:4e/2:0, 16:2e/4:0), LPC (22:4, 20:2, 18:1), and LPE (22:6, 20:4, 20:1, 20:3, 18:1, 18:0), were significantly reversed in the HFD-induced NALFD mice after FFDZ treatment. Numerous studies have reported that phosphatidylcholine (PC) and phosphatidylethanol (PE), as components of phospholipid metabolism, play an important role in regulating lipid and energy metabolism (Veen et al., 2017). Inhibition of PC biosynthesis or a decrease in the PC/PE ratio could further promote obesity-related disease progression through a HFD (Jacobs et al., 2010; Veen et al., 2016). Furthermore, under inflammatory conditions, lysophospholipids are typically hydrolyzed by phospholipase A2 (PLA2), which catalyzes the hydrolysis of PC and PE into LPC and LPE, thereby inducing hepatocyte injury (Yuzbashian et al., 2023). Similar results were also found in our study that PC (20:4e/2:0, 16:2e/4:0), LPC (22:4, 20:2, 18:1), and LPE (22:6, 20:4, 20:1, 20:3, 18:1, 18:0) were positively correlated with body weight, liver weight, IL-6, TNF-α, AST, ALT, and MDA, and negatively correlated with SOD and GPx. Additionally, LPC (22:4, 20:2, 18:1) and LPE (22:6, 20:4, 20:1, 20:3, 18:1, 18:0) were positively correlated with INS, HOMA-IR, FBG, and AUC. Nevertheless, previous studies have also found that the content of LPC (18:1) and LPE (18:1, 18:0) in the livers of mice with HFD-induced lipid metabolism disorders were elevated, thereby leading to insulin resistance (Han et al., 2011; Yaligar et al., 2014). Overall, the results suggest that FFDZ can alleviate the imbalance in lipid homeostasis in NAFLD mice by reversing the abnormal changes in serum lipid metabolites.

In this study, liver transcriptomics profiling of the HFD-induced NAFLD mice following treatment with FFDZ showed that alterations in lipid synthesis and fatty acid oxidation were the most enriched pathways affected by the FFDZ intervention. Disordered expression of lipid synthesis and fatty acid oxidation-related genes induced by HFD were the leading cause of the progression of NAFLD in prior research (Zhi et al., 2022). Therefore, reversing the lipid metabolism disorder in the liver might be the primary pathway through which FFDZ exerts an improvement effect on NAFLD. Consistent with the changes in the results of liver transcriptomics, FFDZ treatment obviously decreased the expression of fatty synthesis-related proteins such as Acaca, Scd-1, Srebp-1c (Srebf1), Acly, and Fasn (Tian et al., 2023), while upregulated the expression of Ppar-α and Cpt-1, which are key regulators associated with fatty acid oxidation (Lefere et al., 2020; Francque et al., 2021). Previous studies have shown that a HFD can activate the sterol regulatory element-binding protein 1c (Srebp-1c), which is the crucial transcription factor involved in *de novo* lipid synthesis, further leading to an increase in the expression of lipid synthesis-related proteins, including Fasn, Acaca, Scd-1, and Acly (Bertolio et al., 2019). Moreover, we examined the expression levels of Ampk and p-Ampk of the liver in HFD-induced NAFLD mice following treatment with FFDZ, based on the results of KEGG pathway enrichment analyses. And the Western blot results exhibited that FFDZ intervention significantly upregulated the expression of p-Ampk in NAFLD mice while decreasing the levels of Ampk. Previous researchers have demonstrated that Ampk, an energy switch, can enhance lipid degradation by up-regulating the Ppar-α and Cpt-1 protein expression, and inhibit *de novo* lipid synthesis through down-regulating the Srebp-1c (Smith et al., 2016; Herzig and Shaw, 2018), indicating FFDZ may improve lipid metabolism disorders by up-regulating p-Ampk expression. In addition, FFDZ decreased the expression of Cd36, which is the key protein that caused lipid deposition in the liver (Rada et al., 2020), suggesting FFDZ prevented the lipid from entering hepatocytes by inhibiting the expression of Cd36. Furthermore, the FFDZ drug serum also significantly reduced the lipid accumulation and the TG content in the oleic acid-induced lipid deposition cell model, which is consistent with the effects observed in NAFLD mice. These above results suggest that FFDZ can reduce liver lipid accumulation in HFD-fed mice by activating the Ampk pathway to inhibit the *de novo* lipid synthesis pathway and enhance fatty acid *β*-oxidation.

Moreover, we identified several active metabolites (Kaempferol, Luteolin, Quercetin, Isorhamnetin, Baicalein, Acacetin, Neobaicalein, and Isorhyncophylline) of FFDZ and their potential roles in lipid metabolism, while the exact contribution of each metabolite to the overall therapeutic effect of FFDZ remains to be elucidated. Subsequent studies should focus on isolating and testing individual metabolites to determine their specific mechanisms of action. Additionally, the study primarily focused on the AMPK signaling pathway and lipid metabolism. Still, NAFLD is a multifactorial disease involving other pathways such as inflammation, oxidative stress, and gut-liver axis interactions. Future research should explore the effects of FFDZ on these additional pathways to provide a more comprehensive understanding of its therapeutic potential.

## 5 Conclusion

**FIGURE 9:**
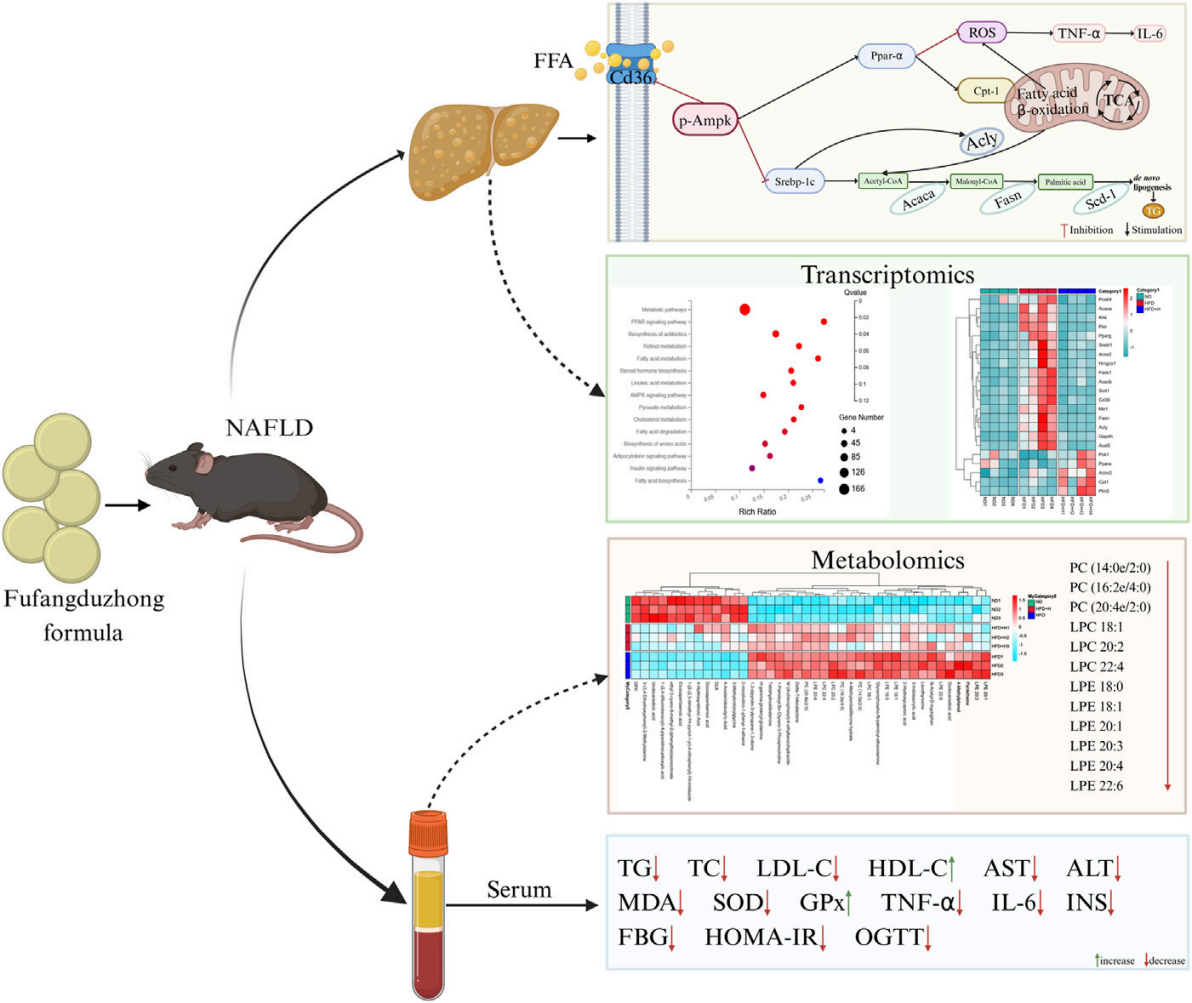
Schematic diagram showing the mechanism of the improved effect of FFDZ on NAFLD.

In general, we demonstrated that FFDZ can remarkedly exert anti-NAFLD effects. Its mechanisms might be associated with the following ameliorative effects on NAFLD that FFDZ exhibits (Figure 9): (1) FFDZ significantly reduced liver damage, glucose intolerance, insulin resistance, the content of harmful lipid metabolites (LPC and LPE), antioxidant defense function injury, and the inflammatory response caused by lipotoxicity in HFD-induced NAFLD mice; (2) FFDZ can reduce lipid accumulation in the liver by activating the Ampk signaling pathway, inhibiting the expression of *de novo* lipid synthesis-related proteins, and increasing the expression of fatty acid *β*-oxidation-related proteins, thereby ameliorating lipid metabolism disorders and hepatic steatosis. Our study initially explored the mechanism of action of FFDZ, which exerts an ameliorative effect on NAFLD caused by HFD and provided a better understanding of FFDZ for the treatment of NAFLD in clinical practice.

In summary, the study’s findings have significant implications for the development of novel treatments for NAFLD, contribute to the growing body of evidence supporting the use of TCM in modern medicine, and provide a methodological blueprint for future research in this area. This will benefit the existing knowledge by offering new insights into the treatment of NAFLD and potentially other metabolic disorders, ultimately improving patient outcomes.

## Data Availability

The data presented in the study are deposited in the NCBI BioProject repository, accession number PRJNA1231032.
