# Supplementary material for: Unraveling the molecular mechanisms of Fufangduzhong formula in alleviating high-fat diet-induced non-alcoholic fatty liver disease in mice
Source: Front Pharmacol. 2025 Mar 12;16:1542143. doi: 10.3389/fphar.2025.1542143 (PMC11936930; doi:10.3389/fphar.2025.1542143)
Supplement: Supplementary file 1 [file DataSheet1.doc]

**Supplementary**

**Materials and methods**

- 1. *UPLC- MS/MS analysis*

The chemical composition of the FFDZ formula and serum containing the drug was analyzed by the UPLC-MS/MS method. 200 μl of FFDZ-containing serum was aspirated and mixed with 800 μl of pre-cooled methanol and left on ice for 5 min. The supernatant was collected by centrifugation at 12,000 rpm for 15 min at 4 °C and dried. The supernatant was then redissolved in 50 μl of methanol and centrifuged at 12,000 rpm for 15 min to collect the supernatant for analysis. Accurately weigh 5 mg of FFDZ formula, add 1 ml of methanol solution, and ultrasonically filter to obtain 5 mg/ml of sample solution. Then, dilute this solution to prepare a 500 μg/ml solution for testing. Each standard was accurately weighed 2 mg, dissolved using methanol and mixed and diluted to a final concentration of 500 ng/ml, 250 ng/ml, 125 ng/ml, 62.5 ng/ml, 31.25 ng/ml, 15.625 ng/ml, 7.8125 ng/ml, 3.90625 ng/ml, 1.953125 ng/ml, 0.9765625 ng/ml, 0.48828125 ng/ml, 0.244140625 ng/ml and 0.1220703125 ng/ml standard solutions.

The prepared solutions were analyzed and identified on an Agilent 1290 Infinity II chromatograph (Santa Clara, CA, USA) and a Sciex Triple Quad ™ 5500+ mass spectrometer (Foster, CA, USA) using a UPLC-MS/MS system. The mobile phase A was 0.1% aqueous acetic acid, mobile phase B was acetonitrile, and the chromatographic column was Titank C18 (2.1 mm×100 mm) with a flow rate of 0.3 ml/min at 35 ℃. The electrospray ionization source was used at a temperature of 500 ℃, and the spray voltage was 5.5 kV for positive ion mode and 4.5 kV for negative ion mode. The ion source gas 1 was 50, the auxiliary pressure was 50, and the collision energy was 34 eV; multiple reaction detection (MRM) modes.

- 1. *Screening for active components and target genes*

TCMSP database (<https://old.tcmsp-e.com/tcmsp.php>) was used to obtain the active ingredients of five traditional Chinese medicines in FFDZ, including *Eucommia ulmoides* Oliv.*, Leonurus artemisia* (Lour.) S. Y. Hu*, Prunella vulgaris* Linn.*, Uncariarhynchophylla* (Miq.) Miq. ex Havil.*, and Scutellaria baicalensis* Georgi (the screening conditions were OB ≥ 30%, and DL ≥ 0.18). Target prediction of active ingredients was performed by the TCMSP database and Swiss Target Prediction database ([http://swisstargetprediction.ch](http://swisstargetprediction.ch/)). Target names were obtained using the UniProt database (https://www.uniprot.org). All the targets were merged and the duplicate values were removed to obtain the target of action of the active ingredients of FFDZ.

- 1. *NAFLD target identification*

The keywords "Non-Alcoholic Fatty Liver Disease" were used to search for potential targets of NAFLD through the Online Mendelian Inheritance in Man database (OMIM) ([https://www.omim.org](https://www.omim.org/)), the Human Gene database (GeneCards) (https://www.genecards.org), and the DisGeNET database (http://disgenet.cn). The GeneCards database selected targets with a relevance score ≥ 23.78. All targets were combined, and duplicates were removed to obtain NAFLD targets.

- 1. *Prediction of FFDZ targets for NAFLD treatment and construction of protein-protein interaction (PPI) network*

The obtained FFDZ active ingredient action targets and NAFLD targets were made into a Venn diagram (<https://jvenn.toulouse.inra.fr/app/index.html>) to get the intersecting genes and screen out the potential action targets of the FFDZ formula for the treatment of NAFLD. Then the drug and active ingredient, disease, and potential target were established into an information network, which was inputted into Cytoscape 3.9.1 software for visualization and analysis to obtain the drug-disease-gene network diagram.

Next, these potential targets were entered into the String database ([https://string-db.org](https://string-db.org/)) for the construction of the PPI network, and the species was Homo sapiens, the minimum interaction score was 0.4, and the free targets were removed. Then the obtained protein interaction results were saved in TSV format and imported into Cytoscape 3.9.1 software, and the network plug-in CytoNCA was used to filter the core targets, which were sorted by Degree value and visualized.

- 1. *Functional enrichment and pathway enrichment analysis*

To explore the possible molecular mechanism of FFDZ in treating NAFLD, these potential gene targets were imported into the DAVID 6.8 database ([https://david.ncifcrf.gov](https://david.ncifcrf.gov/)), set the identifier as OFFICIAL-GENE-SYMBOL limited the species to Homo sapiens, set the threshold *p*＜0.05, and carried out the GO functional enrichment analysis and KEGG pathway enrichment analysis.

**Table S1.** Differential metabolite peak areas identified by UPLC-Q-TOF/MS.

| Metabolite | ND | | | HFD | | | HFD + L | | |
| --- | --- | --- | --- | --- | --- | --- | --- | --- | --- |
| PC (18:2/18:2) | 6.69E+09 | 6.62E+09 | 6.35E+09 | 2.97E+09 | 2.84E+09 | 2.51E+09 | 6.03E+09 | 5.17E+09 | 5.51E+09 |
| PC (18:1/20:5) | 7.43E+08 | 7.05E+08 | 7.01E+08 | 5.43E+08 | 4.64E+08 | 5.65E+08 | 5.35E+08 | 6.25E+08 | 7.55E+08 |
| PC (18:0/20:5) | 1.16E+09 | 1.12E+09 | 1.19E+09 | 6.83E+08 | 5.36E+08 | 7.35E+08 | 1.08E+09 | 1.05E+09 | 1.10E+09 |
| PC (18:1/18:1) | 7.07E+09 | 7.37E+09 | 6.75E+09 | 5.41E+09 | 4.96E+09 | 4.99E+09 | 6.74E+09 | 6.00E+09 | 6.36E+09 |
| PC (20:4e/2:0) | 1.42E+06 | 1.58E+06 | 1.75E+06 | 4.34E+06 | 4.21E+06 | 4.19E+06 | 2.84E+06 | 3.95E+06 | 3.86E+06 |
| PC (16:2e/4:0) | 1.16E+07 | 1.03E+07 | 1.05E+07 | 1.26E+07 | 1.33E+07 | 1.23E+07 | 7.77E+06 | 8.90E+06 | 1.04E+07 |
| PC (14:0e/2:0) | 4.10E+09 | 4.24E+09 | 4.23E+09 | 4.31E+09 | 4.47E+09 | 4.39E+09 | 3.20E+09 | 3.53E+09 | 3.54E+09 |
| PE (18:0/20:5) | 8.05E+05 | 1.57E+06 | 1.18E+06 | 1.33E+06 | 1.90E+06 | 3.13E+06 | 1.12E+06 | 8.58E+05 | 1.23E+06 |
| PE (18:2/18:2) | 1.57E+07 | 1.69E+07 | 1.07E+07 | 1.98E+07 | 1.00E+07 | 2.71E+07 | 1.29E+07 | 1.04E+07 | 1.31E+07 |
| LPC 22:4 | 5.33E+06 | 6.51E+06 | 6.81E+06 | 2.47E+07 | 2.21E+07 | 2.58E+07 | 1.37E+07 | 1.67E+07 | 1.62E+07 |
| LPC 22:6 | 9.75E+08 | 1.10E+09 | 1.04E+09 | 1.23E+09 | 1.21E+09 | 1.28E+09 | 1.18E+09 | 1.16E+09 | 9.05E+08 |
| LPC 20:2 | 3.35E+07 | 3.23E+07 | 2.96E+07 | 4.63E+07 | 5.84E+07 | 5.68E+07 | 2.67E+07 | 3.41E+07 | 2.71E+07 |
| LPC 18:0 | 2.07E+09 | 1.99E+09 | 1.73E+09 | 2.94E+09 | 3.10E+09 | 2.14E+09 | 1.86E+09 | 2.15E+09 | 1.63E+09 |
| LPC 18:1 | 1.91E+09 | 1.83E+09 | 2.00E+09 | 2.55E+09 | 2.27E+09 | 2.14E+09 | 1.75E+09 | 1.95E+09 | 1.73E+09 |
| LPE 20:4 | 3.57E+08 | 4.30E+08 | 5.12E+08 | 1.03E+09 | 1.11E+09 | 9.77E+08 | 6.08E+08 | 6.30E+08 | 7.31E+08 |
| LPE 22:6 | 1.63E+08 | 2.14E+08 | 1.74E+08 | 2.64E+08 | 2.90E+08 | 2.34E+08 | 1.85E+08 | 1.75E+08 | 2.00E+08 |
| LPE 20:1 | 4.09E+06 | 5.06E+06 | 5.48E+06 | 7.56E+06 | 6.99E+06 | 6.64E+06 | 2.45E+06 | 3.46E+06 | 3.67E+06 |
| LPE 20:3 | 2.74E+07 | 1.62E+07 | 2.18E+07 | 3.84E+07 | 4.18E+07 | 3.44E+07 | 1.80E+07 | 1.82E+07 | 1.75E+07 |
| LPE 18:0 | 9.86E+07 | 1.17E+08 | 1.58E+08 | 3.65E+08 | 3.03E+08 | 2.41E+08 | 1.34E+08 | 1.29E+08 | 1.64E+08 |
| LPE 18:1 | 9.74E+07 | 1.21E+08 | 1.46E+08 | 2.13E+08 | 1.88E+08 | 1.85E+08 | 9.13E+07 | 1.06E+08 | 1.00E+08 |
| Arachidonic acid | 1.60E+09 | 2.03E+09 | 2.49E+09 | 4.00E+09 | 3.55E+09 | 3.45E+09 | 2.98E+09 | 2.47E+09 | 2.68E+09 |


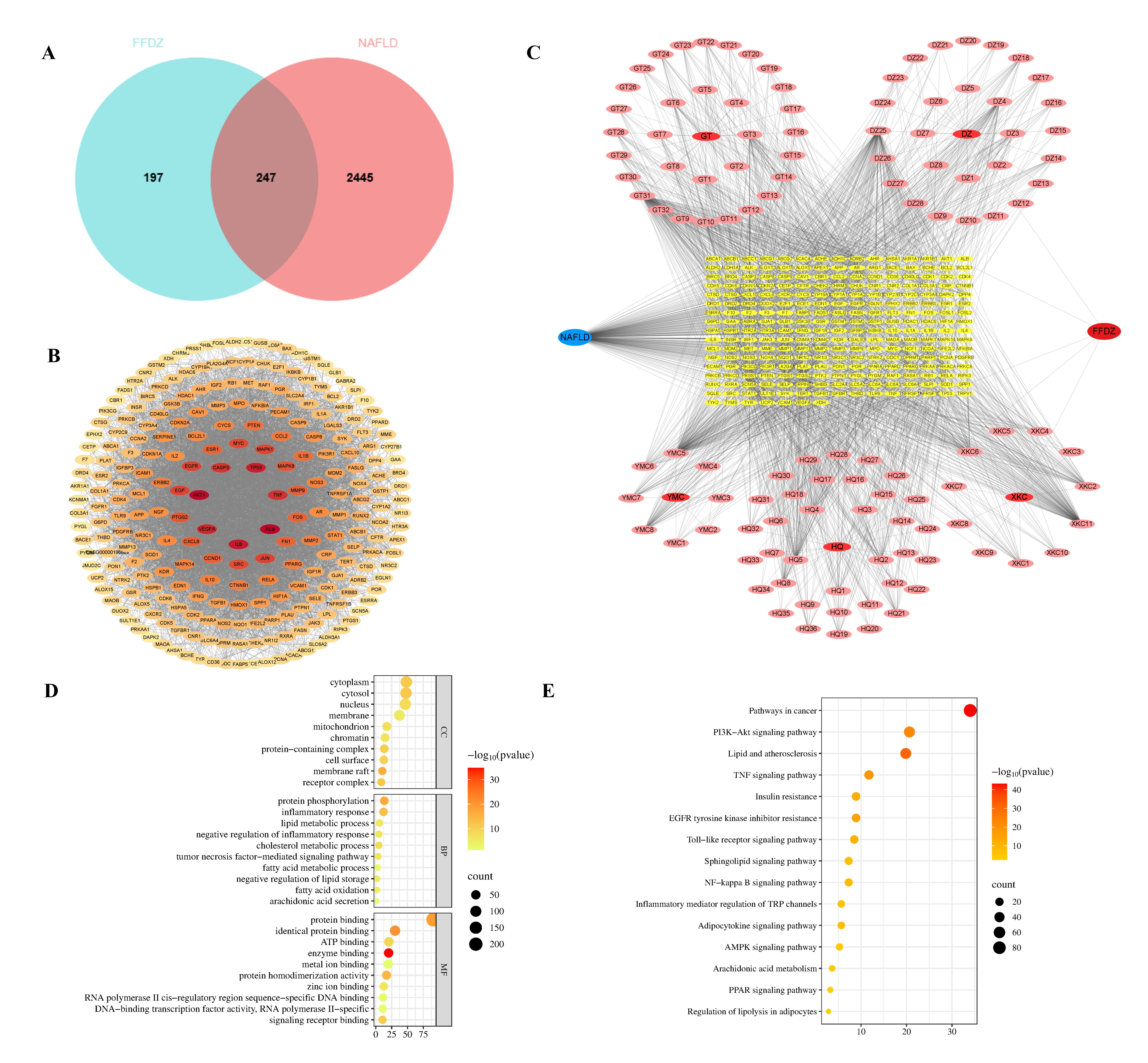


**Fig. S1.** Network pharmacology analysis. (A) Venn diagram of FFDZ and NAFLD related targets; (B) PPI network diagram of FFDZ for treating NAFLD. The higher the degree value of the target, the darker the color, and the more nodes; (C) Drug-disease-gene network relationship diagram; (D) GO enrichment analysis of potential FFDZ targets; (E) KEGG enrichment analysis.
